# Do generic measures fully capture health-related quality of life in adult, general critical care survivors?

**DOI:** 10.1186/cc13201

**Published:** 2014-03-17

**Authors:** W Lim, N Black, K Rowan, N Mays

**Affiliations:** 1 London School of Hygiene & Tropical Medicine, London, UK; 2 Intensive Care National Audit & Research Centre, London, UK

## Introduction

We examined the extent to which the two generic health- related quality of life (HRQL) measures recommended for use in adult, general critical care [1] - the SF-36 and EQ-5D - captured survivors' HRQL, which is important in assessing the effectiveness of critical care. Unlike other fields of healthcare that employ both generic and specific HRQL measures, most recent studies in critical care have used only generic measures, despite uncertainty as to their appropriateness.

## Methods

A patient-based conceptual framework for survivors' HRQL was built using: a systematic review of the literature; secondary analysis of 40 in-depth interviews with adult critical care survivors; and primary analysis of in-depth interviews with a maximum variation sample of 25 white critical care survivors in England. Two methods were then used to assess the extent to which the SF-36 and EQ-5D captured their HRQL, as detailed in the framework: the content of both instruments was examined, alongside data collected from the in-depth interviews; and the opinions that survivors expressed about how accurately the SF-36 and EQ-5D reflected their ideas on health and HRQL were analysed and taken into account.

## Results

The patient-based framework was in two parts: the first covered survivors' personal status which consisted of their physical status, emotional/psychological status and cognitive status; and the second comprised nine subdomains affected by their personal status. The latter were: activities and behaviours; physical zone of comfort and/or activity; interactions and relationships with others; perception of, interpretation of, and responses to life; personality; external appearance; suitability and availability of clothes; place of residence; and finances. The current generic measures recommended on the basis of expert consensus for use with survivors of adult, general critical care have substantial gaps in their coverage of this conceptual framework for survivors' HRQL, particularly in relation to their cognitive status and its subsequent impact.

## Conclusion

The two most commonly used generic HRQL measures in adult, general critical care have significant gaps in their coverage of survivors' HRQL. Any (new) critical care-specific HRQL measure should be designed specifically to capture the impact of critical illness on survivors' cognitive status and its subsequent effects.
